# RCSB Protein Data Bank 1D3D module: displaying positional features on macromolecular assemblies

**DOI:** 10.1093/bioinformatics/btac317

**Published:** 2022-05-11

**Authors:** Joan Segura, Yana Rose, Sebastian Bittrich, Stephen K Burley, Jose M Duarte

**Affiliations:** Research Collaboratory for Structural Bioinformatics Protein Data Bank, San Diego Supercomputer Center, University of California, La Jolla, CA 92093, USA; Research Collaboratory for Structural Bioinformatics Protein Data Bank, San Diego Supercomputer Center, University of California, La Jolla, CA 92093, USA; Research Collaboratory for Structural Bioinformatics Protein Data Bank, San Diego Supercomputer Center, University of California, La Jolla, CA 92093, USA; Research Collaboratory for Structural Bioinformatics Protein Data Bank, San Diego Supercomputer Center, University of California, La Jolla, CA 92093, USA; Research Collaboratory for Structural Bioinformatics Protein Data Bank, Rutgers, The State University of New Jersey, Piscataway, NJ 08854, USA; Institute for Quantitative Biomedicine, Rutgers, The State University of New Jersey, Piscataway, NJ 08854, USA; Department of Chemistry and Chemical Biology, Rutgers, The State University of New Jersey, Piscataway, NJ 08854, USA; Cancer Institute of New Jersey, Rutgers, The State University of New Jersey, New Brunswick, NJ 08901, USA; Research Collaboratory for Structural Bioinformatics Protein Data Bank, San Diego Supercomputer Center, University of California, La Jolla, CA 92093, USA

## Abstract

**Motivation:**

Mapping positional features from one-dimensional (1D) sequences onto three-dimensional (3D) structures of biological macromolecules is a powerful tool to show geometric patterns of biochemical annotations and provide a better understanding of the mechanisms underpinning protein and nucleic acid function at the atomic level.

**Results:**

We present a new library designed to display fully customizable interactive views between 1D positional features of protein and/or nucleic acid sequences and their 3D structures as isolated chains or components of macromolecular assemblies.

**Availability and implementation:**

https://github.com/rcsb/rcsb-saguaro-3d.

**Supplementary information:**

Supplementary data are available at *Bioinformatics* online.

## 1 Introduction

Mapping positional features from 1D sequences onto 3D structures of biological macromolecules facilitates interrogation of relationships between shape and function. Sequence to structure mapping enables identification of spatial correlations and geometric patterns among protein or nucleic acid annotations that would be obscured if they were analyzed solely using linear polymer sequences. Over the last few years, various libraries, web applications and software tools have been developed to compute alignments among structures, and protein and gene sequences, and visualize positional features over the different levels of molecular organization from genome to macromolecular assemblies. 3DBIONOTES (Segura *et al.*, 2017), for example, is a stand-alone web application that integrates biochemical annotations from multiple resources and visualizes them at 1D sequence and 3D structure levels. Similarly, MolArt (Hoksza *et al.*, 2018) is a JavaScript library that integrates and visualizes UniProt (UniProt Consortium, 2021) annotations with protein structural data. Finally, the RCSB PDB 1D coordinate server (Segura *et al.*, 2021) provides alignments and mapping of annotations between genome and protein sequence resources, including structures of macromolecular assemblies.

In this work, we present a new TypeScript/JavaScript module designed to create custom interactive views between 1D sequence positional features and 3D structures of macromolecules over the web. The main motivation behind this development is to provide the structural bioinformatics community with a flexible and fully customizable tool that can be adapted for use in various contexts. To achieve this end, the library exposes multiple event callbacks that allow software developers to define bidirectional interactions between 1D positional features and 3D atomic coordinates of experimental structures (from PDB) or computed structure models [from AlphaFold2 (Jumper *et al.*, 2021) or RoseTTAFold (Baek *et al.*, 2021), etc.]. Moreover, it allows arrangement of positional features in multiple sequence viewers and defining many-to-many relationships between 3D structure information and 1D viewers (see Supplementary Fig. S1). The module was built atop the open-source Mol* Viewer (Sehnal *et al.*, 2021) and the RCSB PDB Feature Viewer (Segura *et al.*, 2021). Currently, the library is used at the RCSB PDB rcsb.org web portal (Burley *et al.*, 2021) to display a bidirectional interactive view of mappings between sequence annotations and 3D macromolecular structures.

## 2 Materials and methods

The RCSB PDB 1D3D module is an open-source library written in TypeScript that is designed to visualize interactive views between 1D positional features and 3D biostructures. The library comprises a collection of React (https://reactjs.org/) components that integrate the Mol* Viewer and the RCSB PDB Feature Viewer (see Supplementary Section S1). 1D positional features and 3D structures are rendered in separate components that communicate with each other when external events (clicking or hovering) occur. These events trigger a set of configurable callback functions that define how 1D features and 3D atomic coordinates interact. Moreover, the 1D and 3D viewers APIs are accessible from the event callback functions, allowing modification of viewer content or representation of displayed elements.

### 2.1 Structure component

The structure component integrates the Mol* Viewer for the 3D visualization of macromolecular structures. The component configuration tool allows choices as to how structure data is loaded. The exposed loading configuration accepts different types of parameters, including individual or multiple PDB IDs, a URL pointing to a computed structure model from resources such as AlphaFoldDB (Varadi *et al.*, 2021) or the ModelArchive (Schwede *et al.*, 2009), or a plain string encoding the 3D structure information. In addition, the configuration includes multiple options to modify the Mol* graphical user interface. (See Supplementary Section S2 for a detailed description of the structure component configuration interface.)

### 2.2 Sequence component

The sequence component integrates the RCSB PDB Feature Viewer. This component is responsible for displaying the 1D positional features and encoding the logic that enables interoperability between 1D features and 3D structure information. Positional features are organized in two levels. First, a specific feature viewer allocates a collection of features as part of its configuration. Second, multiple feature viewers can be grouped into different blocks (see Supplementary Fig. S1). Thus, the sequence component contains a collection of blocks, wherein each block encodes the configuration for one or more feature viewers, including associated 1D positional features. Feature viewers belonging to the same block are displayed simultaneously. However, only a single block can be activated at any given time. The sequence component configuration includes different mechanisms to define how blocks can be activated or deactivated (see Supplementary Section S3).

Interaction of positional features and 3D structures is configured through different callback functions that are triggered when mouse click or hover events occur on 3D structures or 1D features. These functions are defined at the feature viewer level. Hence, each feature viewer in each block may implement its own event callback configuration. When an event (mouse click or hover) occurs within the structure component, callback functions are triggered as defined in all the feature viewers belonging to the active block. Event data and all relevant information needed to identify the relevant polymer component (i.e. amino acid or nucleotide) or ligand, including requisite identifiers, are passed to the callback as state parameters. Thereafter, based on callback parameter information, each feature viewer determines whether to process or ignore the call. For a detailed description of the interoperation configuration between sequence and structure components see Supplementary Section S3.

## 3 Summary

Herein, we present RCSB Protein Data Bank 1D3D module, a novel open-source library designed for visualizing interactive environments between 1D positional features and 3D structures of biological macromolecules. The library exposes a rich and flexible configuration interface that allows developers to define interoperation between multiple 1D positional feature viewers and multiple 3D atomic coordinate models.

The library is publicly available in github and published as an npm module. It is easy to install and reusable within any web resource. Currently, the RCSB PDB rcsb.org web portal uses this tool to display an interacting mapping between 1D protein features and the 3D structures of biomolecules.

## Funding

This work was supported by the National Science Foundation [DBI-1832184]; the US Department of Energy [DE-SC0019749]; and the National Cancer Institute, National Institute of Allergy and Infectious Diseases and National Institute of General Medical Sciences of the National Institutes of Health [R01GM133198] (Principal Investigator: Stephen K. Burley).

*Conflict of Interest*: none declared.

## Supplementary Material

btac317_Supplementary_Data
