# Supplementary material for: RCSB Protein Data Bank 1D3D module: displaying positional features on macromolecular assemblies
Source: Bioinformatics. 2022 May 11;38(12):3304–5. doi: 10.1093/bioinformatics/btac317 (PMC9191206; doi:10.1093/bioinformatics/btac317)
Supplement: btac317_Supplementary_Data [file btac317_supplementary_data.docx]

**RCSB Protein Data Bank 1D3D module: displaying positional features on macromolecular assemblies**

Joan Segura, Yana Rose, Sebastian Bittrich, Stephen K. Burley and Jose M. Duarte

**S1 RCSB Protein Data Bank 1D3D module configuration**

The RCSB PDB 1D3D module is an open-source library designed to visualyze interactive environments between 1D positional features and 3D structural models of macromolecular assemblies. The library comprises a collection of React modules that integrates the Mol* plugin (<https://github.com/molstar/molstar>) and the RCSB Protein Data Bank (PDB) Feature Viewer (<https://github.com/rcsb/rcsb-saguaro>) for the visualization of 3D models and 1D features, respectively. Structural models and positional features are rendered in two separate React components: the structure component and the sequence component (see Figure S1). These two components communicate with each other through different callback functions that are triggered when hover or click events occur in 3D structural models or 1D positional features. The logic behind these callbacks functions is configurable through the sequence component properties. In addition, this component configuration also encodes the information of the 1D positional features and their visualization arrangement. The structure component provides the configuration to load the 3D structural models and different parameters to configure the Mol* Graphical User Interface (GUI).


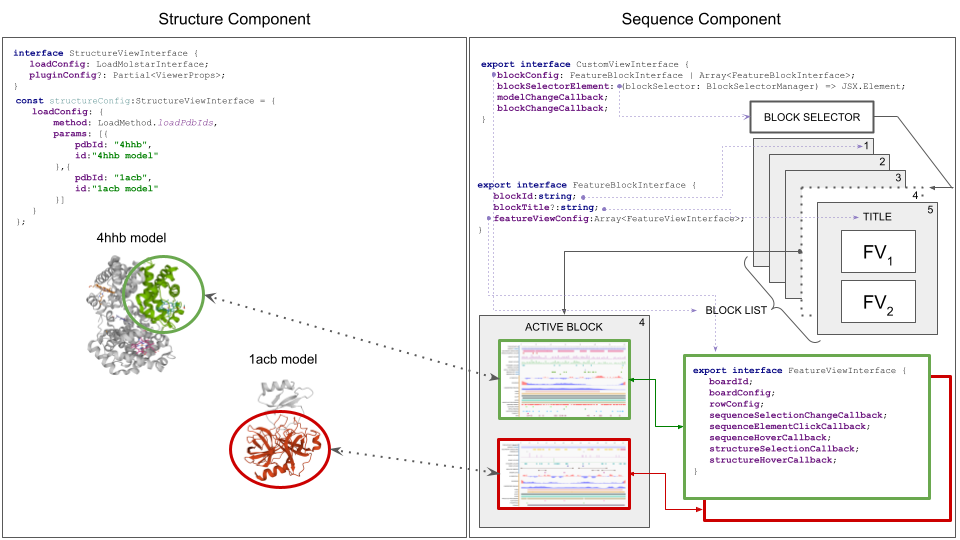


**Figure S1 Structure and sequence component configuration interface.** Configuration schema of the Structure (left) and Sequence (right) components. In this example, the Structure component loads two different 3D models; PDB codes 4hhb and 1acb, respectively. The sequence panel configures multiple blocks with multiple 1D feature viewers. Block number 4, the current active block and visible, defines two feature viewers each of them interacting with different chains of the 3D models. The feature viewer highlighted in green iis linked to the 4hhn model chain B (displayed in green color) and the feature viewer highlighted in red interacts with the 1acb model chain A (displayed in red color).

The configuration of sequence and structure components is accessible from the properties of the root component (see Table S1). In addition to the structure and sequence configuration, an optional parameter (**cssConfig**) to configure the style and arrangement of the structure and sequence HTML containers is also available. Sequence and structure components are rendered in a pair of HTML “div” elements under the same root container (a third “div” element). The “**cssConfig**” includes different attributes to configure the styles for the root and the pair of contained elements. Finally, the “**elementId**” parameter identifies the HTML element where the whole 1D3D component is rendered.

**Table S1 1D3D Root component configuration interface**

| **class** RcsbFv3DCustom **extends** RcsbFv3DAbstract {  **constructor**(config: RcsbFv3DCustomInterface);  ...  } |
| --- |
| **interface** RcsbFv3DCustomInterface **extends** RcsbFv3DAbstractInterface {  **structurePanelConfig**: RcsbFvStructureInterface;  **sequencePanelConfig**: {  **config**: CustomViewInterface;  **title**?: **string**;  **subtitle**?: **string**;  };  } |
| **interface** RcsbFv3DAbstractInterface {  **elementId: string;**  **cssConfig?:** RcsbFv3DCssConfig**;**  } |

**S2 Structure component configuration**

The structural component provides the mechanisms to load the 3D models and to configure the Mol* Viewer GUI. The loading configuration includes the method and parameters that identifies what models are loaded: PDB Ids, URLs or a plain string encoding the 3D structural information (see Table S2, “LoadMolstarInterface”). Additionally, if a 4x4 rotation-translation matrix is provided (see Table S2, “LoadParams”) the loaded model will be transformed accordingly for its visualization. Finally, the string parameter “**id**“ (see Table S2, “LoadParams”) can be used to identify the models when interactivity callbacks functions are defined.

**Table S2 Structure component configuration interface**

| **interface** RcsbFvStructureInterface {  **loadConfig**: LoadMolstarInterface;  **pluginConfig**?: Partial<ViewerProps>;  } | |
| --- | --- |
| **interface** LoadMolstarInterface {  **loadMethod**: LoadMethod;  **loadParams**: LoadParams \| Array<LoadParams>;  } | |
| **enum** LoadMethod {  ***loadPdbId*,**  ***loadPdbIds*,**  ***loadStructureFromUrl*,**  ***loadSnapshotFromUrl*,**  ***loadStructureFromData***  } | **interface** LoadParams {  **pdbId?: string;**  **url?: string,**  **data?: string \| number[],**  **matrix?: *Mat4*;**  **id?:string;**  } |

The structure component configuration also includes a parameter (**pluginConfig**) to configure the Mol* plugin GUI (see Annex Table S6).

**S3 Sequence component configuration**

The sequence component encodes the configuration of how 1D positional features are arranged and how they interact with the 3D structural models. Positional features are organized in two levels: a collection of positional features is rendered by a particular feature viewer and multiple viewers can be grouped in different blocks. The feature viewer belonging to the same block will be displayed simultaneously and only a single block can be activated at the same time. The module provides the mechanisms to configure how blocks can be activated or deactivated (see next).

Table S3 displays the different parameters needed to configure the sequence component. The parameter “**blockConfig**” encodes the configuration for one or more blocks (see Table S4). When multiple blocks are defined, the parameter “**blockSelectorElement**” must be used to provide the mechanism to select and change the active block. This parameter is a function that returns a React element. The returned element will be rendered as part of the sequence component (for instance a HTML select dropdown button) and used as part of the GUI to change the active block. The input of this function is a “BlockSelectorManager” object (see Annex Table S7) that is used to select the active block using the “**blockId**” (see Table S4). When a new block is activated all the feature viewers defined in its configuration will be displayed and any other block feature viewer will be hidden.

**Table S3 Structure component configuration interface**

| **interface** CustomViewInterface {  **blockConfig**: FeatureBlockInterface \| Array<FeatureBlockInterface>;  **blockSelectorElement**?: (bsm: BlockSelectorManager) => JSX.Element;  **blockChangeCallback**?: (  plugin: SaguaroPluginPublicInterface,  pfvList: Array<RcsbFv>,  selection: RcsbFvSelectorManager) => **void**;  **modelChangeCallback**?:  (modelMap: SaguaroPluginModelMapType) => CustomViewStateInterface;  } |
| --- |

In addition to “**blockSelectorElement**”, the parameter “**blockChangeCallback**” defines a callback function that will be triggered when the active block changes. The callback parameters include the object that handles the Mol* Viewer, a list of the active feature viewer objects and the selection state object (see Annex Table S8). The last configuration argument “**modelChangeCallback**” is an optional callback function that will be called when a new 3D model is loaded or removed from the Mol* display. The parameter of this callback function (SaguaroPluginModelMapType) is a map that identifies the currently loaded 3D models with their associated chains. The return type “CustomViewStateInterface” contains the same fields described in Table S3 omitting the parameter “**modelChangeCallback**”. This allows reconfiguring the blocks and feature viewers when new models are loaded or removed from the Mol* Viewer.

Each sequence component block encodes the configuration for one or more feature viewers (see Table S4 “**featureViewConfig**” parameter). The “**blockId**” parameter is a string that identifies a particular block and can be used to change and select the active block through a “BlockSelectorManager” object (see Table S3 and Annex Table S7).

**Table S4 Block configuration interface**

| **interface** FeatureBlockInterface {  **blockId**:**string**;  **featureViewConfig**: Array<FeatureViewInterface> \| FeatureViewInterface;  } |
| --- |

The feature viewer configuration (Table S5) contains the information of the 1D positional features that will be displayed in the viewer (**rowConfig**), the parameters needed to render the RCSB PDB Feature Viewer (**boardConfig**) and different callbacks functions that implement the interactivity between the 1D features and the 3D models.

**Table S5 Feature viewer configuration interface**

| **interface** FeatureViewInterface {  **boardId**?:**string**;  **boardConfig**: RcsbFvBoardConfigInterface;  **rowConfig**: Array<RcsbFvRowConfigInterface>;  **sequenceSelectionChangeCallback**: (  plugin: SaguaroPluginPublicInterface,  selectorManager: RcsbFvSelectorManager,  sequenceRegion: Array<RcsbFvTrackDataElementInterface>) => **void**;  **sequenceElementClickCallback**: (  plugin: SaguaroPluginPublicInterface,  selectorManager: RcsbFvSelectorManager,  d: RcsbFvTrackDataElementInterface) => **void**;  **sequenceHoverCallback**: (  plugin: SaguaroPluginPublicInterface,  selectorManager: RcsbFvSelectorManager,  hoverRegion: Array<RcsbFvTrackDataElementInterface>) => **void**;  **structureSelectionCallback**: (  plugin: SaguaroPluginPublicInterface,  fv: RcsbFv,  selectorManager: RcsbFvSelectorManager) => **void**;  **structureHoverCallback**: (  plugin: SaguaroPluginPublicInterface,  fv: RcsbFv,  selectorManager: RcsbFvSelectorManager) => **void**;  } |
| --- |

These callback functions are triggered when click or hover events occur in the 3D models or the 1D positional features. When a click or hover event occurs in a 3D structural model, the event associated callback function is triggered for all feature viewers that belong to the current active block. The callback function parameters include the object that handles the Mol* plugin and the selection state object (see Annex Tabel S8). When events occur in the structure component, the feature viewer object is included in the callback parameters. On the other hand, when callbacks are triggered due sequence component events, information on the hovered, clicked or selected region is included in the callback parameters. The callback functions are triggered with the following events:

- **sequenceSelectionChangeCallback** is triggered when the RCSB PDB Feature Viewer selected region changes including changes from the viewer API
- **sequenceElementClickCallback** is triggered when a 1D feature element is clicked
- **sequenceHoverCallback** is triggered when the mouse hovers the feature viewer or any of the rendered 1D feature elements
- **structureSelectionCallback** is triggered when any region of a 3D model is selected
- **structureHoverCallback** is triggered when a 3D model is hovered

**Annex**

The Mol* Viewer GUI configuration interface provides a set of fields to configure which controls are available. Table S6 lists the possible options.

**Table S6 Mol* GUI configuration interce**

| **type** ViewerProps {  **showImportControls**: **boolean**;  **showExportControls**: **boolean**;  **showSessionControls**: **boolean**;  **showStructureSourceControls**: **boolean**;  **showSuperpositionControls**: **boolean**;  **layoutIsExpanded**: **boolean**;  **layoutShowControls**: **boolean**;  **layoutShowSequence**: **boolean**;  **layoutShowLog**: **boolean**;  **viewportShowExpand**: **boolean**;  **viewportShowSelectionMode**: **boolean**;  **backgroundColor**: Color;  **showWelcomeToast**: **boolean**;  }; |
| --- |

The block state interface defines a collection of methods that can be used to update or collect the current active block. When a new active block is updated the feature viewers defined in its configuration will be rendered and become visible (see Section S3).

**Table S7 Block state interface**

| **interface** BlockSelectorManager {  setActiveBlock(blockId:**string**): **void**;  getActiveBlock(): **string**;  getPreviousBlock(): **string**;  } |
| --- |
| (blockSelectorManager: BlockSelectorManager) => {  **return** (  **<div>**  **<select onChange=**{(e)=>{  blockSelectorManager**.setActiveBlock(**e.**target.value)**  }}**>**  **<option value={"blockId_1"}>**Block 1**</option>**  **<option value={"blockId_2"}>**Block 2**</option>**  **</select>**  **</div>**  );  } |

The selection state interface defines a collection of functions designed to keep track of which 1D sequence and/or 3D model regions that have been selected thro click of hover events.

**Table S8 Selection state interface**

| **interface** RcsbFvSelectorManagerInterface {  setSelectionFromRegion(  modelId: **string**,  labelAsymId: **string**,  region: RegionSelectionInterface,  mode:'**select**'\|'**hover**');  addSelectionFromRegion(  modelId: **string**,  labelAsymId: **string**,  region: RegionSelectionInterface,  mode:'**select**'\|'**hover**');  getSelection(mode:'**select**'\|'**hover**'): Array<ChainSelectionInterface>;  clearSelection(mode:'**select**'\|'**hover**'): **void**;  } |
| --- |
